# Clinician, patient, and carer views on neuromodulation for epilepsy: is there rationale for a randomised controlled trial of VNS vs. DBS?

**DOI:** 10.1016/j.bas.2026.106055

**Published:** 2026-04-17

**Authors:** Shobana Chandrashekar, Michelle Ruiz-Perez, Rory J. Piper, Aswin Chari, Michael G. Hart

**Affiliations:** a Developmental Neuroscience, Great Ormond Street Institute of Child Health, University College London, London, UK; b School of Health & Medical Sciences, City St George's, University of London, Cranmer Terrace, SW17 0RE, UK

**Keywords:** Drug-resistant epilepsy, Neuromodulation therapies, Survey data, Comparative study, Clinical trial design, Patient perspectives

## Abstract

**Introduction:**

This study aimed to gather UK clinician, patient, and carer perspectives on vagus nerve stimulation (VNS) and deep brain stimulation (DBS) to inform the design of clinical trials comparing these treatments for drug-resistant epilepsy (DRE).

**Research question:**

To assess stakeholder views on these treatments and their willingness to participate in a clinical trial comparing VNS and DBS for DRE.

**Methods:**

Two surveys were conducted: one for clinicians in neurology, neurophysiology, and neurosurgery, and another for patients/carers. The estimated response rates were 2.7% and 0.008% for the surveys respectively.

**Results:**

Among the 32 clinician responses all agreed VNS has a role in treating DRE, as did 94% for DBS. While 84% felt VNS had sufficient evidence for routine use, only 23% said the same for DBS. Median minimum age for treatment consideration was 5 years for VNS and 8 years for DBS. Clinicians agreed on the proposed trial's scientific validity (median Likert score 4/5) and interest in efficacy comparison (5/5); 72% would refer patients for the trial. Of the 38 patient/carer responses, 13% had VNS. Around one-third would consider VNS (32%) or DBS (34%). Concerns included procedure anxiety, side effects, and battery changes. While 53% were willing to enter the proposed trial, 84% preferred to choose their treatment.

Key outcomes identified included seizure reduction, seizure freedom, quality of life, and SUDEP risk.

**Discussion and conclusion:**

Clinician, patient, and carer insights highlight the need for robust evidence on VNS and DBS efficacy. These findings will guide future trials in managing DRE.

## Highlights

• Surveyed clinicians see value in a VNS vs. DBS trial.
• Patients consider seizure control and quality of life as key trial outcomes.
• Concerns about VNS/DBS include surgery risks, side effects, and battery changes.
• Over half of surveyed patients (52.6%) would consider joining a trial.
• Findings inform future neuromodulation research and trial design.

## 1 Introduction

Despite many new antiseizure medications (ASM) in the last 30 years, the proportion of patients with drug-resistant epilepsy (DRE) remains steady at approximately 30%, which highlights the unmet need for non-pharmacological interventions (Benbadis et al., 2018).

Vagus nerve stimulation (VNS) is an established therapeutic intervention, with landmark trials such as the E03 and E05 trials establishing efficacy (Musselman et al., 2019). Notably, VNS efficacy appears to increase over time. (Dibué-Adjei et al., 2019; Englot et al., 2011; Orosz et al., 2014; Ryzí et al., 2013).

Conversely, deep brain stimulation (DBS) is an emerging option approved by the FDA in 2018 for adults with epilepsy (Vetkas et al., 2022). The SANTE (Stimulation of the Anterior Nucleus of the Thalamus in Epilepsy) trial and the ESTEL (Electrical Stimulation of Thalamus for Epilepsy of Lennox–Gastaut phenotype) trial are pivotal studies that established the efficacy of DBS in reducing seizure frequency in patients with focal epilepsy and Lennox Gastaut syndrome respectively (Fisher et al., 2010), (Dalic et al., 2022).

The National Institute for Health and Clinical Excellence (NICE) recommendation (2020) for DBS for DRE in adults varies depending on the site of stimulation. Clinicians should perform ANT-DBS (DBS targeting the anterior nucleus of the thalamus or ANT) only with special arrangements for clinical governance, patient consent, and thorough auditing or research, owing to limited evidence. For other targets, they should undertake such procedures exclusively within the context of research, given the lack of sufficient evidence. (Guidance NICE).

Currently, direct comparative data between VNS and DBS is limited, with much of the data drawn from single-centre, retrospective studies. One recent study (Zhu et al., 2021) reported that ANT-DBS provided a greater reduction in seizure frequency than VNS over a 12-month follow-up period, while another (Alcala-Zermeno et al., 2022) highlighted variations in seizure reduction across different neurostimulation techniques and suggested that cortical stimulation strategies may offer some advantages over subcortical options, including ANT-DBS and VNS.

Given the lack of robust comparative data, clinician and patient perspectives are crucial in assessing the readiness for wider adoption of DBS and identifying patient-centred priorities in treatment design. Clinician perspectives help gauge medical community acceptance of DBS, reflecting their confidence and willingness to support this innovative intervention. Similarly, insights from patients and caregivers illuminate the real-world impact of these treatments, capturing essential feedback on quality of life, perceived benefits, and logistical challenges. This feedback is invaluable for designing patient-centred clinical trials, ensuring studies address relevant clinical endpoints and quality of life outcomes (Faulkner et al., 2023).

The proposed DoVE Trial (DBS or VNS for Epilepsy) is a randomised controlled trial comparing the efficacy of VNS and DBS for DRE. This study aims to gather perspectives from clinicians, patients with epilepsy, and their caregivers to inform such a trial's design and development.

As part of this study, surveys were conducted to gather perspectives on neuromodulation for DRE from key stakeholder groups. These included UK medical consultants treating epilepsy, as well as individuals with epilepsy and their carers. The aim was to capture both professional and patients’ personal views on the use of neuromodulation as a treatment option and assess interest in participating in the proposed DoVE trial.

By gathering insights through surveys this study aims to contribute to the optimisation of epilepsy treatment strategies, serving as a guide to the development of the DoVE trial which will establish the roles for DBS and VNS in the management of DRE.

## 2 Methods

Two online surveys were conducted. Both consisted of a mix of question types, including single best answer, multiple answer, Likert scales and free text answers. PDF copies of the two surveys are included in the Supplementary material.

### 2.1 Clinician survey

A piloted online survey was designed and distributed via online channels and mailing lists of relevant specialty associations including the Society of British Neurological Surgeons, the Association of British Neurologists, the British Society of Clinical Neurophysiology, the British Society for Stereotactic and Functional Neurosurgery, the British Branch of the International League Against Epilepsy and the British Paediatric Neurology Association. The survey was likely sent out only once by each society/group. It was stipulated that only consultants in (adult or paediatric) neurology, neurophysiology or neurosurgery were eligible to complete the survey. Survey responses were collected between December 2023 and February 2024.

The survey had 4 sections:

Section 1: Demographic Information.

Section 2: Views on role of VNS for DRE.

Section 3: Views on role of DBS for DRE.

Section 4: Views on a potential UK multi-centre randomised trial of DBS and VNS.

### 2.2 Patient/carer survey

A piloted online survey, with an embedded video (link included in the PDF copy of the patient/carer survey, in the Supplementary material) explaining the treatments and a proposed trial to compare the two, was distributed to the patient/carer e-mail lists of relevant associations within the UK, including the Epilepsy Research Institute's SHAPE network, Young Epilepsy, and Epilepsy Action. The survey was distributed between January and March 2024, likely only once by each society/group.

The survey had 4 sections:

Section 1: A short video explaining current and future treatments for epilepsy.

Section 2: Questions about the person with epilepsy.

Section 3: Views about current and future stimulation treatments for epilepsy.

Section 4: Views about the proposed DoVE trial.

### 2.3 Data analysis

Descriptive reporting of summary statistics and data visualisation of survey responses were conducted on R Studio and Microsoft Excel. Bar charts summarise categorical data, Likert plots illustrate scaled responses from 1 to 5, and the raincloud plot depicts the distribution of clinician-reported minimum ages for neuromodulation therapies. The raincloud plot combines a half-violin (distribution), a box plot (medians and interquartile ranges), and a dot plot (individual responses).

## 3 Results

The clinician survey received 32 responses, and the patient/carer survey received 38 responses. Whilst a response rate for both surveys is not accurately able to be calculated, we estimate 1200 eligible clinicians (800 neurologists, 300 neurosurgeons, 100 neurophysiologists) and 500,000 persons with epilepsy. This would result in an estimated response rate of 2.7% for the clinician survey and 0.008% for the patient/carer survey.

### 3.1 Clinician survey

#### 3.1.1 Demographics (section 1)

Of the 32 clinicians, 13 (40.6%) specialized in neurosurgery, 13 (40.6%) in neurology, and 6 (18.8%) in neurophysiology. Almost half of the clinicians (43.8%) had 10-12 years of consultant experience, 37.5% had under 10 years, and 18.8% had over 20 years. Responses were from across the UK, with Greater London (25%), South East (21.9%), and Yorkshire and Humber (12.5%) being the most represented regions. The complete demographic characteristics of the clinicians are detailed in Table 1.

**Table 1:** Demographics of Clinicians, n = 32.

Demographic variable	Response	n (%)
Speciality	Neurosurgery	13 (40.6%)
	Neurology	13 (40.6%)
	Neurophysiology	6 (18.8%)
Number of years as a consultant	<10 years	12 (37.5%)
	10-20 years	14 (43.8%)
	>20 years	6 (18.8%)
Region of the UK working in	Greater London	8 (25%)
	South East	7 (21.9%)
	Yorkshire and the Humber	4 (12.5%)
	East of England	3 (9.4%)
	Scotland	3 (9.4%)
	South West	3 (9.4%)
	North West	2 (6.3%)
	West Midlands	1 (3.1%)
	Wales	1 (3.1%)
	Northern Ireland	0
	East Midlands	0
	North East	0

#### 3.1.2 Sections 2-4

**Fig. 1:**
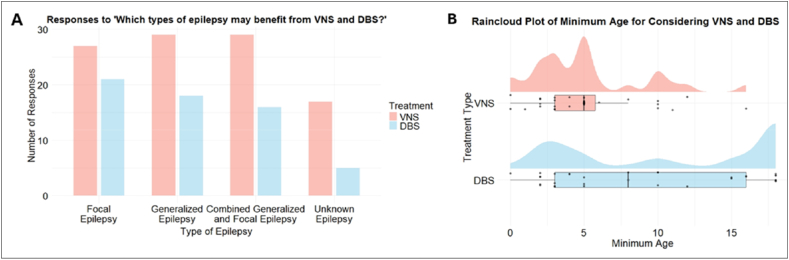
Clinician (n = 32) views on neuromodulation as a treatment for drug-resistant epilepsy. A, paired bar chart shows the count of responses on the potential benefit of VNS and DBS for various epilepsy types, allowing a comparison between the two treatments. B, raincloud plot displays the distribution of clinician-reported minimum ages for considering VNS and DBS.

All clinicians (100%) agreed that VNS has a role in the treatment of DRE and 30 (93.8%) agreed on DBS's role. When considering what types of epilepsy may benefit from VNS, more than 84.4% of respondents thought it would be beneficial for focal and generalized epilepsy. Clinicians seem to think DBS is less beneficial overall, with 70% agreeing that it is beneficial for focal epilepsy (Fig. 1A).

Majority of respondents (68.8%) considered 5 years or younger as the minimum age to consider VNS as a treatment, with the median age (and modal age) being 5 years, while this was quite varied for DBS with an interquartile range of minimum age of 13 years, median of 8 years and modal age of 18 years (Fig. 1B).

According to the survey, 84.4% of clinicians believed there is currently enough evidence to support the routine use of VNS, while only 22.6% said the same for DBS, as a treatment for DRE. With regards to VNS, respondents commented that the absence of randomised controlled trial data is a significant gap, and while there are ongoing studies, evidence remains stronger for focal seizures compared to generalized seizures. For DBS, respondents said the evidence is still developing and there is a clear need for data from a randomised clinical trial.

**Fig. 2:**
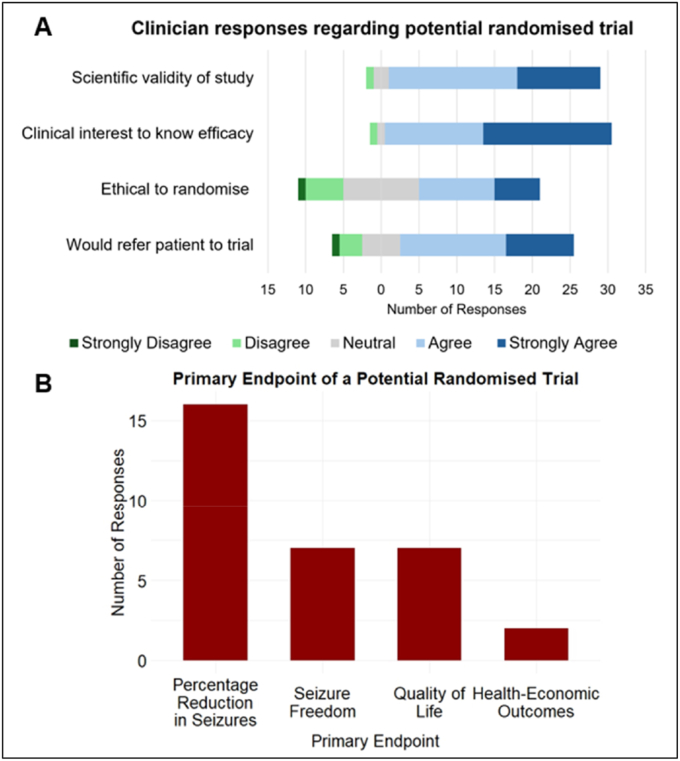
Clinician (n = 32) views on potential randomised trial. A, Likert plot summarises clinician opinions on aspects of the potential trial design and shows the overall sentiment regarding the proposed trial. B, bar chart shows the clinician responses regarding the preferred primary endpoint for the trial and highlights which endpoints are considered most important by clinicians when evaluating the trial's success.

The clinicians were asked about their views on a potential study, a UK multi-centre randomised trial of VNS and DBS. They were asked to report their opinions about the design of the trial on a Likert scale of 1-5, illustrated as a Likert scale plot (Fig. 2A). Clinicians agreed that the potential trial has scientific validity (median = agree; 87.5% agreed/strongly agreed), is of clinical interest (median = strongly agree; 93.7% agreed/strongly agreed), and they would refer patients to it (median = agree; 71.9% agreed/strongly agreed), while the median response for whether it was ethical to randomise was neutral (31.3% neutral and 50.1% agreed/strongly agreed).

The preferred trial endpoint was seizure reduction (50%), followed by seizure freedom and quality of life (both 21.9%) (Fig. 2B).

### 3.2 Patient/carer survey

#### 3.2.1 Demographics (section 2)

In the patient/carer survey, 71.1% were persons with epilepsy while the rest were from a carer for someone with epilepsy. Participants were evenly distributed across age groups, with Greater London (23.7%) being the most represented region. Just over a quarter (26.3%) of the patients have a learning disability. The data also shows that 7.9% of the persons with epilepsy have had resective surgery for epilepsy and 13.2% have a VNS device. The complete demographic characteristics of the survey respondents are detailed in Table 2. The survey did not include questions on if they had offered or been evaluated for epilepsy surgery.

**Table 2:** Demographics of Patients/Carers, n = 38.

Demographic variable	Response	n (%)
Age group	0-19	10 (26.3%)
	20-39	10 (26.3%)
	40-59	10 (26.3%)
	60+	8 (21.2%)
Region of the UK living in	Greater London	9 (23.7%)
	South West	7 (18.4%)
	Soth East	5 (13.2%)
	North West	5 (13.2%)
	Wales	3 (7.9%)
	East Midlands	3 (7.9%)
	North East	2 (5.3%)
	Yorkshire and the Humber	2 (5.3%)
	West Midlands	1 (2.6%)
	Scotland	1 (2.6%)
	East of England	0
	Northern Ireland	0
Persons with epilepsy that have had any form of surgery for epilepsy	Yes	3 (7.9%)
	No	35 (92.1%)
Persons with epilepsy that have a VNS	Yes	5 (13.2%)
	No	33 (86.8%)

#### 3.2.2 Sections 3-4

**Fig. 3:**
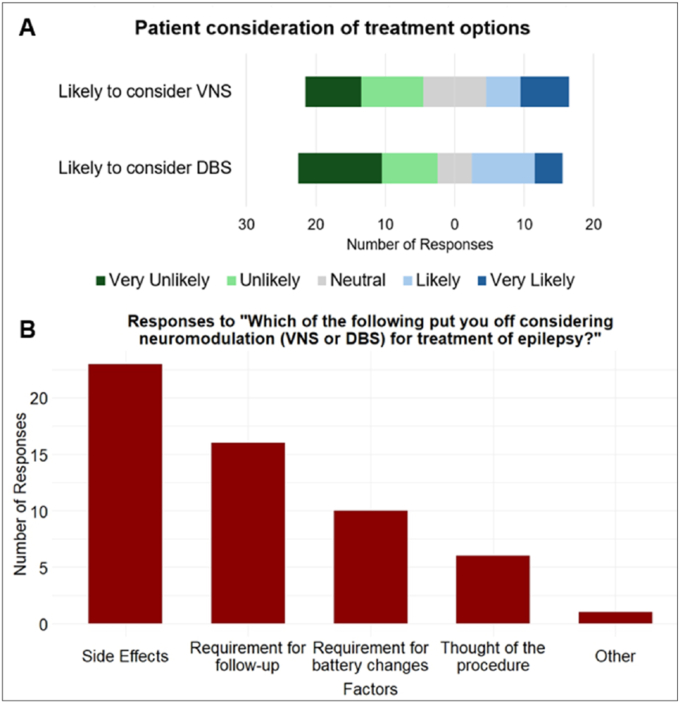
Patients' (n = 38) consideration of VNS or DBS as treatment options. A, Likert plot illustrates the likelihood of patients to consider VNS or DBS as a treatment option and indicates a higher likelihood of patients considering VNS compared to DBS. B, bar chart presents factors that deter patients from considering neuromodulation as a treatment option and highlights the prevalence of each factor affecting patient decision-making.

The proportion of respondents likely to consider VNS stands at 31.6%, and 34.2% show a likelihood to consider DBS (Fig. 3A).

The key deterrents are shown in Fig. 3B. Some other factors under the graphed category ‘Other’ (in Fig. 3B) entered as comments by respondents were- ‘Epilepsy isn't severe enough to undergo treatment’ and ‘Inability to come off some of current medication’.

**Fig. 4:**
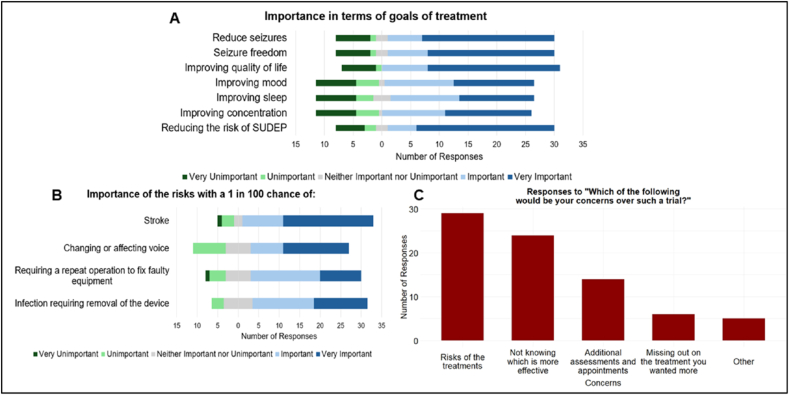
Patient (n = 38) views on a potential randomised trial. A, Likert plot illustrates patient opinions on the importance of treatment goals and provides insight into which is considered most critical by patients. B, Likert plot displays patient views on the importance of various risks associated with the potential trial, each with a 1 in 100 chance of occurring; it illustrates how patients weigh the significance of these risks. C, bar chart illustrates concerns related to the potential trial and the frequency of the concerns reported by patients.

Patients identified improving quality of life (81.6%) as the highest-priority treatment goal, followed by reducing SUDEP risk, achieving seizure freedom, and reducing seizures (all 76.3%) (Fig. 4A). Improving concentration and mood (both 68.4%) and improving sleep (65.8%) were also considered important.

The survey stated that risks of VNS and DBS are slightly different as one is an operation in the neck and the other is an operation on the brain. Fig. 4B shows how important patients/carers would consider the risks. Patients/carers were informed in the survey that VNS requires battery changes every 2-4 years, which is usually performed as a day case operation under general anaesthesia. Some DBS systems offer the option of being able to wirelessly recharge the battery, which can last up to 30 years. The modal response was 5 (very important) on the Likert scale of 1 to 5, with 73.7% of respondents considering this difference between the two treatments important.

About half (52.6%) of the respondents were willing to enter the proposed DoVE trial if they or a family member would be referred. Some of the reasons for interest in participation included wanting to try VNS/DBS before resective surgery as it is a less invasive option, improving quality of life and improve the understanding of epilepsy treatments, wanting to try a method besides medications (that have not led to seizure freedom), and the exhaustion of all other options. The main reasons why they would not want to participate in the trial were the thought of ‘being used as a guinea pig’, fear of surgery, fear of side effects, and the discomfort of being randomly assigned into one of the treatments.

Majority (73.7%) of the respondents agreed that if the person with epilepsy has a learning disability, it would be ethical to get permission to participate in the study from their carer or next-of-kin. These surveyors commented that the carer or next-of- kin making the decision would be safer. Others said that both the person with epilepsy with a learning disability and their carer or next-of-kin, should be involved in making this decision.

The most common concern (76.3%) regarding the trial were the risks of the treatments**.** Nearly two-thirds (63.2%) of patients/carers expressed concerns about not knowing which treatment is more effective and/or about missing their preferred option (Fig. 4C). The majority (84.2%) would prefer to be offered both treatments and choose which to proceed with.

Additionally, 15.8% were concerned about frequent assessments and travel burdens (travel and convenience cost). Half (50%) of the respondents thought that a visit every 3 months would be most convenient, while 26.3% thought a visit once a month would be appropriate.

**Fig. 5:**
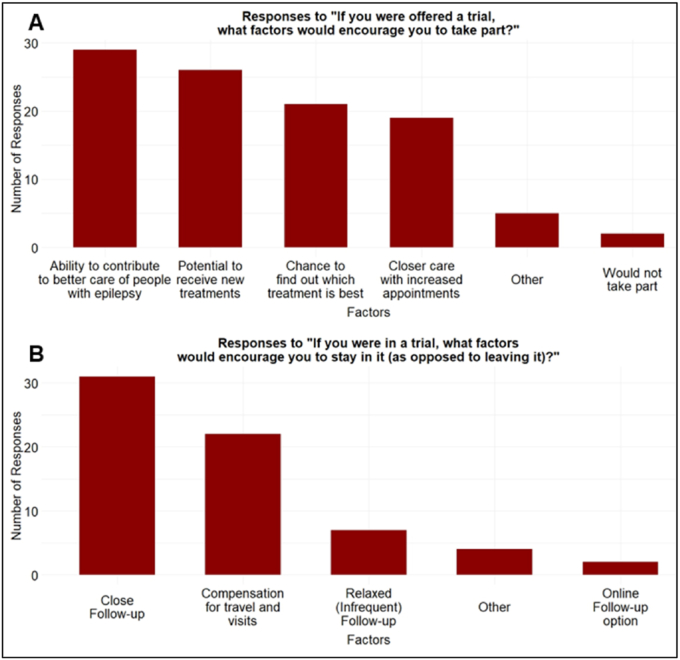
Patient interest to participate in a potential randomised trial. A, bar chart shows patient responses regarding factors that would encourage their participation in a trial and provides insights into incentives for patient enrolment. B, bar chart shows patient responses regarding factors that would encourage them to stay in the trial rather than withdraw and provides insights into aspects that influence patient retention.

Patients and their carers identified factors that would encourage them to participate in the trial if it were offered to them. Most (78.4%) wanted to contribute to better epilepsy care, 70.3% were motivated by access to new treatments, and 56.8% would take part to help find out which treatment is best. Nearly half (48.6%) would be encouraged by the closer care they would receive (Fig. 5A). Among ‘Other’ factors were “Possibility of reducing number of medications taken,” “Recognition by the team of the psychological impacts on individuals participating as well as the neurological effects”, and “Potential to stop all seizures and end side effects from medication”.

It was then important to learn what factors would encourage potential trial participants to stay in the trial. Patients and their carers picked close follow-up (83.8%), travel compensation (59.5%), and relaxed or infrequent follow-up (18.9%) as the top factors (Fig. 5B). Two respondents suggested that follow-up appointments be a hybrid of video calls and in-person visits.

## 4 Discussion

The survey results offer a selective overview of both clinician and patient/carer perspectives on neuromodulation for DRE in the UK, making the case for further studies to compare and optimise these treatments. The results, however, must be interpreted cautiously due to the extremely low response rates relative to total number of eligible clinicians and patients with epilepsy in the UK.

Clinicians prioritised seizure reduction as the primary outcome but also emphasised seizure freedom and quality of life. While clinicians expressed willingness to participate in the proposed trial by referring patients, there appears to be a discord between clinicians' willingness to contribute to the trial and their views on the ethics of randomisation, potentially highlighting complex challenges that could arise if the trial were to move forward. On the patient and carer side, DBS was more likely to be considered compared to VNS, but overall, about half expressed a willingness to participate in the trial. Key reasons for trial participation included the exhaustion of other treatment options, altruism, and the hope of achieving meaningful treatment goals.

VNS is widely used in children and adults (Fukuda et al., 2024), whereas DBS, though promising in adults, lacks randomised controlled trial evidence in paediatrics (Khan et al., 2021). Surveyed clinicians had varied opinions on minimum age for DBS, showing greater caution than with VNS, stemming from limited evidence and potential risks, particularly in children. These gaps underscore the need for targeted trials, to clarify DBS's role and optimise outcomes for this demographic (Yan et al., 2018).

Our findings of clinician views on primary outcomes relates to the metrics seen in neuromodulation trials (Kuba et al., 2009), (Elliott et al., 2011), (Vale et al., 2011), (Ching et al., 2013) and aligns with expert opinions highlighted in Kaufmann et al., that outcome evaluation should extend beyond seizure metrics to include neurocognitive function, mood, and quality of life (Kaufmann et al., 2020). Kaufmann et al. noted that ‘optimal outcome is only achieved if patient expectations are met besides significant improvement in clinical parameters’ (Kaufmann et al., 2020). This suggests that any future trials, must account for a range of clinical and patient-centred outcomes to comprehensively evaluate treatment efficacy.

Patient/carer-reported key treatment goals closely align with the literature, which identifies at least seven distinct goals of epilepsy treatment: to reduce seizure frequency and severity, to improve function, to enhance quality of life, to promote coping, to improve external circumstances, to prevent premature death, and (in children) to promote growth and development (Brülde and Malmgren, 1999). Balzekas et al. (2024) provide insights into patient and carer priorities, highlighting that neuromodulation therapies are often viewed as a ‘last remaining option’ after failure of ASMs. Patients/carers prioritise improving quality of life, reducing seizures, and regaining independence, even without guaranteed seizure freedom. However, the study also reveals notable concerns about the risks and invasiveness of DBS, including potential complications such as stroke and infection, which temper patient enthusiasm (Balzekas et al., 2024). Despite these apprehensions, potential improvements drive willingness, underscoring the need to manage expectations and safety concerns.

Our study also reveals significant concerns that could affect trial participation, including the perceived surgical risks, and uncertainty about which treatment option would be more effective. DBS involves higher perioperative risks compared to VNS, with complications such as intracranial haemorrhage (4.5% of implants), infections (12.7% at 10 years), and lead misplacement (8.2%) (Simpson et al., 2022). Serious complications, such as tract haemorrhage, occur in less than 5% of cases, and mortality is very rare. DBS may also have preliminary associations with mood and memory effects, although most adverse effects are transient and related to stimulation parameters (Benbadis et al., 2018). In contrast, VNS has a lower risk profile, with complications typically like superficial infections (3–6%) and sometimes infections requiring device removal, vocal cord paralysis, and bradycardia/asystole (Kuba et al., 2009), (Elliott et al., 2011), (Simpson et al., 2022), (Gooneratne et al., 2016). Unique risks include hoarseness and cough from recurrent laryngeal nerve involvement and potential effects on sleep apnoea, though permanent risks are less than 3% (Benbadis et al., 2018), (Kuba et al., 2009), (Elliott et al., 2011), (Ching et al., 2013), (Gooneratne et al., 2016). While neuromodulation devices are generally safe, issues like lead breakage and other device-related issues remain concerns (Benbadis et al., 2018), (Gooneratne et al., 2016). A significant concern with ANT-DBS reported in Salanova et al. was a gradual worsening in neuropsychological composite scores over time, with increased depression, anxiety, and total mood disturbance (Gooneratne et al., 2016), (Salanova et al., 2015). Despite these issues, improvements were observed in attention, executive function, and subjective cognitive function, suggesting some cognitive benefits alongside the adverse outcomes (Gooneratne et al., 2016), (Salanova et al., 2015). These differences in surgical risks and potential complications between VNS and DBS may influence patient decisions and their willingness to participate in trials.

Patients and carers expressed a strong preference for choosing between VNS and DBS over randomisation, reflecting a shared concern with Balzekas et al.'s findings about maintaining autonomy in treatment decisions. Allowing patient choice would introduce significant bias, highlighting the challenge of conducting an unbiased clinical trial comparing VNS and DBS. Addressing these concerns will be critical to ensuring the success of potential comparative studies. Providing detailed information, managing patient expectations, and offering thorough counselling can help overcome these barriers. Reflecting to the clinicians’ view on treatment randomisation, transparency and thorough counselling will be essential to build confidence and encourage participation from both clinicians and patients. These findings suggest that beyond the scientific and medical value of the trial, its design must address logistical and practical aspects that affect patient convenience and perceived benefit. In addition, should a randomised controlled trial be undertaken, a consideration of economic evaluation would be important. A trial-based cost-effectiveness analysis could inform the relevance of such a study to broader stakeholders such as NICE.

A key finding from this study is that patients value the battery differences between VNS and DBS. Current VNS systems do not have rechargeable options, necessitating more frequent revisions (Simpson et al., 2022). Furthermore, rechargeable devices depend on patient compliance for optimal performance, as missed recharges can reduce efficacy.

### 4.1 Limitations

The very low response rates raise important concerns about the external validity of the responses. As the surveys were distributed exclusively to individuals affiliated with specific epilepsy charities and societies, the sample may not reflect the broader epilepsy population. Moreover, the survey data is only UK-based and may not capture the global variations in attitudes towards the treatments, concerns regarding them, and the readiness of the medical community to accept such treatments in their regime.

## 5 Conclusion

In conclusion, this study highlights the current clinician and patient perspectives on VNS and DBS, providing a strong rationale for a well-designed randomised controlled trial. The DoVE trial, incorporating patient-centred outcomes and addressing the concerns raised in these surveys, has the potential to contribute significantly to the treatment landscape for drug-resistant epilepsy. By including both efficacy and quality of life as primary outcomes, the trial will align with the needs and preferences of both clinicians, and patients and their carers, offering a comprehensive evaluation of these neuromodulation techniques.

## Funding

AC & RP are supported by NIHR Academic Clinical Lectureships and the NIHR GOSH Biomedical Research Centre.

## Declaration of competing interest

The authors declare that they have no known competing financial interests or personal relationships that could have appeared to influence the work reported in this paper.
