# Supplementary material for: Clinician, patient, and carer views on neuromodulation for epilepsy: is there rationale for a randomised controlled trial of VNS vs. DBS?
Source: Brain Spine. 2026 Apr 17;6:106055. doi: 10.1016/j.bas.2026.106055 (PMC13127170; doi:10.1016/j.bas.2026.106055)
Supplement: Multimedia component 1 [file mmc1.pdf]

# Neuromodulation for Drug-Resistant Epilepsy

This short survey is designed to canvas UK clinician perspectives on neuromodulation including Deep Brain Stimulation (DBS) and Vagus Nerve Stimulation (VNS) for drug-resistant epilepsy, with a view to informing clinical trial development.

The survey has been endorsed by the British Branch of the ILAE and the British Society of Stereotactic and Functional Neurosurgery. UK-based consultants in (adult or paediatric) neurology, neurophysiology and neurosurgery are eligible to complete this survey. It is divided into 4 sections and should take no more than 10 minutes to complete.

By participating, you are agreeing to your anonymised data being used in academic publications and presentations.

* Indicates required question

## Section 1: Demographic Information

1. Which specialty do you belong to? *

*Mark only one oval.*

- Neurosurgery
- Neurology
- Neurophysiology

2. How long have you been a consultant? *

*Mark only one oval.*

- <10 years
- 10 - 20 years
- >20 years

3. Do you routinely treat people with epilepsy? *

*Mark only one oval.*

- Yes
- No

4. Which region of the UK do you work in?

*Mark only one oval.*

- Greater London
- South East
- South West
- West Midlands
- North West
- North East
- Yorkshire and the Humber
- East Midlands
- East of England
- Scotland
- Wales
- Northern Ireland

5. Are any of these services currently available at your centre (not necessarily for epilepsy)?

*Check all that apply.*

- [ ] VNS
- [ ] VNS Specialist Nurse
- [ ] DBS
- [ ] DBS Specialist Nurse

## Section 2: Views on role of VNS for drug-resistant epilepsy

6. Is there a role for VNS in the treatment of drug-resistant epilepsy? *

*Mark only one oval.*

- ( ) Yes
- ( ) No

7. Which types of epilepsy may benefit from VNS?

*Check all that apply.*

- [ ] Focal epilepsy
- [ ] Generalised epilepsy
- [ ] Combined focal & generalised epilepsy
- [ ] Unknown epilepsy

8. What should be the minimum age for considering VNS? *

*Mark only one oval.*

- 0
- 1
- 2
- 3
- 4
- 5
- 6
- 7
- 8
- 9
- 10
- 11
- 12
- 13
- 14
- 15
- 16
- 17
- 18
- Option 14

9. Is there currently enough evidence to support the routine use of VNS for drug-resistant epilepsy? *

*Mark only one oval.*

- Yes
- No

10. Any further comments about the current evidence base?

## Section 2: Views on role of DBS for drug-resistant epilepsy

11. Is there a role for DBS in the treatment of drug-resistant epilepsy? *

*Mark only one oval.*

- ◯ Yes
- ◯ No

12. Which types of epilepsy may benefit from DBS?

*Check all that apply.*

- [ ] Focal epilepsy
- [ ] Generalised epilepsy
- [ ] Combined focal & generalised epilepsy
- [ ] Unknown epilepsy

13. What should be the minimum age for considering DBS? *

*Mark only one oval.*

- 0
- 1
- 2
- 3
- 4
- 5
- 6
- 7
- 8
- 9
- 10
- 11
- 12
- 13
- 14
- 15
- 16
- 17
- 18
- Option 14

14. Is there currently enough evidence to support the routine use of DBS for drug-resistant epilepsy? *

*Mark only one oval.*

- Yes
- No

15. Any further comments about the current evidence base?

_______________________________________________

_______________________________________________

_______________________________________________

_______________________________________________

_______________________________________________

## Section 3: View on a potential UK multi-centre randomised trial of DBS and VNS

We would like your view on a potential study, which would seek to randomise patients with drug-resistant epilepsy to either VNS or DBS. Any patient with focal and/or generalised epilepsy that has been discussed at an epilepsy surgery MDT meeting and not a candidate for focal treatment would be eligible for the study. We envisage that this is the same criteria currently used for referral for Vagus Nerve Stimulation (VNS) treatment.

If eligible, patients (or their carers) may meet with study team members to discuss study enrolment and would be treated with either VNS or DBS based on the treatment allocation.

16. On a scale of 1-5, do you agree that this study has scientific validity? *

*Mark only one oval.*

| | 1 | 2 | 3 | 4 | 5 | |
|---|---|---|---|---|---|---|
| Stro | ◯ | ◯ | ◯ | ◯ | ◯ | Strongly Agree |

17. On a scale of 1-5, is it of clinical interest to know which of these 2 treatments is more efficacious? *

*Mark only one oval.*

| | 1 | 2 | 3 | 4 | 5 | |
|---|---|---|---|---|---|---|
| No i | ◯ | ◯ | ◯ | ◯ | ◯ | Utmost interest |

18. On a scale of 1-5, is it ethical to randomise to VNS or DBS? *

*Mark only one oval.*

| | 1 | 2 | 3 | 4 | 5 | |
|---|---|---|---|---|---|---|
| Higł | ◯ | ◯ | ◯ | ◯ | ◯ | Highly ethical |

19. On a scale of 1-5, would you support such a study by referring your eligible patients? *

*Mark only one oval.*

| | 1 | 2 | 3 | 4 | 5 | |
|---|---|---|---|---|---|---|
| Stro | ◯ | ◯ | ◯ | ◯ | ◯ | Strongly support |

20. Which patients would you refer to such a study? *

*Check all that apply.*

- [ ] Focal epilepsy
- [ ] Generalised epilepsy
- [ ] Combined focal & generalised epilepsy
- [ ] Unknown epilepsy

21. What should be the primary endpoint of such a study? *

*Mark only one oval.*

- ◯ Seizure freedom
- ◯ Percentage reduction in seizures
- ◯ Quality of life
- ◯ Health-economic outcomes
- ◯ Medications
- ◯ Other: ______________________

22. Any other comments?

23. If you would like to be involved in the planning of such a study, please provide your e-mail address. It will stored securely and only used for this specific purpose in line with GDPR.

This content is neither created nor endorsed by Google.

Google Forms
