# Supplementary material for: Clinician, patient, and carer views on neuromodulation for epilepsy: is there rationale for a randomised controlled trial of VNS vs. DBS?
Source: Brain Spine. 2026 Apr 17;6:106055. doi: 10.1016/j.bas.2026.106055 (PMC13127170; doi:10.1016/j.bas.2026.106055)
Supplement: Multimedia component 2 [file mmc2.pdf]

Future Treatments for Epilepsy

Hello!

We are a group of doctors and researchers in London and are keen to understand your perspectives as a person with epilepsy or a carer for a person with epilepsy on current and future treatments. This is to help us design studies to improve future treatments for epilepsy, specifically those that involve electrical stimulation to reduce seizures, which is known as 'neuromodulation'.

This survey will be in 4 sections and should take about 20 minutes to complete.

- **Section 1**: This section is a short video will explain current and future treatments for epilepsy to help inform your answers.
- **Section 2**: This section will ask some questions about you and, if you are a carer, about the person with epilepsy that you care for.
- **Section 3**: This section will ask you some questions about current and future stimulation treatments for epilepsy.
- **Section 4**: This section will ask you some questions about our proposed DoVE trial.

Please note that we will not be collecting any identifiable information about you. We have given an option at the end to provide your e-mail address if you would like to be updated on the results of the survey and will use your e-mail for this purpose only. All data will be stored in compliance with GDPR.

Aswin Chari
University College London

Rory Piper
University College London

Michael Hart
St George's, University of London

* Indicates required question

Section 1: Current and future treatments for epilepsy

Please take a few minutes to watch this video before moving on to the next section.

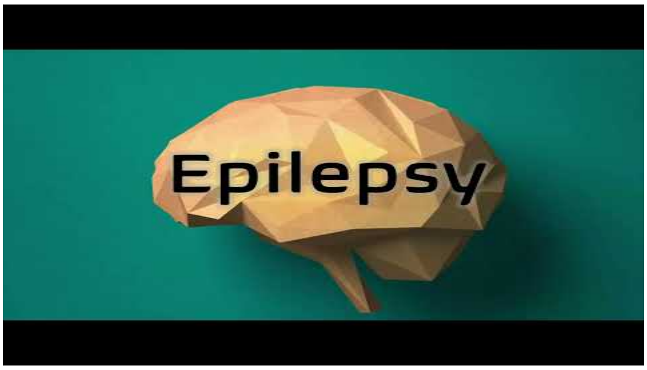

http://youtube.com/watch?v=jVzndd1KJg8

Section 2: Some questions about you

1. Which one of these best describes you? *

Mark only one oval.

- A person with epilepsy
- A carer for someone with epilepsy

The following questions relate to the person with epilepsy (either yourself or the person you care for).

2. What age is the person with epilepsy? *

Mark only one oval.

- 0-19
- 20-39
- 40-59
- 60+

3. Does the person with epilepsy have a learning disability? *

Mark only one oval.

- Yes
- No

4. Which region of the UK does the person with epilepsy live? *

Mark only one oval.

- Greater London
- South East
- South West
- West Midlands
- North West
- North East
- Yorkshire and the Humber
- East Midlands
- East of England
- Scotland
- Wales
- Northern Ireland

5. Has the person with epilepsy had any form of surgery for epilepsy? *

Mark only one oval.

- Yes
- No

6. Does the person with epilepsy have a vagus nerve stimulator (VNS)? *

Mark only one oval.

- Yes
- No

Section 3: Current and future stimulation treatments for epilepsy

7. On a scale of 1-5, how likely are you to consider vagus nerve stimulation (VNS) for treatment of epilepsy? *

Mark only one oval.

	1	2	3	4	5	
Very						Very likely

8. On a scale of 1-5, how likely are you to consider deep brain stimulation (DBS) for treatment of epilepsy? *

Mark only one oval.

	1	2	3	4	5	
Very						Very likely

9. If neuromodulation (DBS or VNS) was deemed a treatment option for your/your caree's epilepsy, how important are the following in terms of the goals of treatment? *

Mark only one oval per row.

	Very unimportant	Unimportant	Neither important nor unimportant	Important	Very important
**Reduce seizures (either frequency or severity)**					
**Stopping seizures completely (seizure freedom)**					
**Improving quality of life**					
**Improving mood**					
**Improving sleep**					
**Improving concentration**					
**Reducing the risk of sudden unexpected death in epilepsy (SUDEP)**					

10. Which of the following put you off considering neuromodulation (DBS or VNS) for treatment of epilepsy?

(You may select more than one response)

Check all that apply.

- [ ] Side effects
- [ ] Requirement for follow-up
- [ ] Requirement for battery changes
- [ ] Thought of the procedure
- [ ] Other: ______________________

11. The risks of DBS and VNS are slightly different as one is an operation on the brain and the other is an operation in the neck. How important would you consider the following risks? *

Mark only one oval per row.

	Very Unimportant	Unimportant	Neither important nor unimportant	Important	Very Important
**A 1 in 100 chance of stroke**	⬭	⬭	⬭	⬭	⬭
**A 1 in 100 chance of changing or affecting voice**	⬭	⬭	⬭	⬭	⬭
**A 1 in 100 chance of requiring a repeat operation to fix faulty equipment**	⬭	⬭	⬭	⬭	⬭
**A 1 in 100 chance of infection requiring removal of the device**	⬭	⬭	⬭	⬭	⬭

12. VNS requires battery changes every 2-4 years, which is usually performed as a day case operation under general anaesthesia. DBS offers the option of being able to wirelessly recharge the battery, which can last up to 30 years. On a scale of 1-5, how important is this difference? *

Mark only one oval.

	1	2	3	4	5	
Not	◯	◯	◯	◯	◯	Very important

Section 4: Views on the DoVE Trial

As mentioned in the video, the DoVE Trial is a study we are proposing to test which of two stimulation treatments - deep brain stimulation (DBS) or vagus nerve stimulation (VNS) - may be better for people with epilepsy.

Usually, when someone does not respond to medication, they undergo tests which determine whether they may be candidates for surgery to help with their epilepsy. Some are offered VNS.

Our current plans are that, if someone is offered VNS, and wishes to participate in DoVE, they will enter the study and randomly assigned to one of the 2 treatments, either DBS or VNS. Following the operation, they will be followed up by the doctors and the research team for 2 years and, at the end of the 2 years, we will see how well each patient is doing. We will monitor their seizures, quality of life and other outcomes whilst also closely assessing how safe and what complications are associated with DBS and VNS respectively.

DBS or VNS for Epilepsy

13. If you or your loved one had been referred for epilepsy surgery, would you be willing to enter our proposed DoVE trial and be randomly assigned to receive either deep brain stimulation (DBS) or vagus nerve stimulation (VNS) as a treatment for epilepsy? *

Mark only one oval.

- ◯ Yes
- ◯ No

14. Please explain why you would or would not participate.

15. If there was the option, would you prefer to be offered both treatments (DBS or VNS) and choose which to proceed with? *

Mark only one oval.

- Yes
- No

16. If the person with epilepsy has a learning disability, would it be ethical to get permission to participate in the study from their carer or next-of-kin? *

Mark only one oval.

- Yes
- No

17. Please explain why you do or do not think this is acceptable.

18. Which of the following would be your concerns over such a trial? *

(You may select more than one response)

Check all that apply.

- [ ] Risks of the treatments
- [ ] Not knowing which is more effective
- [ ] Additional assessments and appointments associated with a trial
- [ ] Missing out on the treatment you wanted more
- [ ] Other: ____________

19. In a trial, we usually assess people at multiple time points to see how they are doing. However, this comes at a travel and convenience cost. How frequent do you think visits should be? *

Mark only one oval.

- Once a month
- Once every 3 months
- Once every 6 months
- Once a year

20. If you were offered a trial, what factors would encourage you to take part?

(You may select more than one response)

Check all that apply.

- Potential to receive new treatments
- Closer care with increased appointments
- Chance to find out which treatment is best
- Ability to contribute to better care of people with epilepsy
- Other: ____________________

21. If you were in a trial, what factors would encourage you to stay in it (as opposed to leaving it)?

(You may select more than one response)

Check all that apply.

- Close follow-up
- Relaxed (infrequent) follow-up
- Compensation for travel and visits
- Other: ____________________

22. Do you have any further comments or questions about VNS, DBS or the DoVE trial?

This content is neither created nor endorsed by Google.

Google Forms
